# Lobbying Expenditures in the US Health Care Sector, 2000-2020

**DOI:** 10.1001/jamahealthforum.2022.3801

**Published:** 2022-10-28

**Authors:** William L. Schpero, Thomas Wiener, Samuel Carter, Paula Chatterjee

**Affiliations:** 1 Division of Health Policy and Economics, Department of Population Health Sciences, Weill Cornell Medical College, New York, New York; 2 Department of Medicine, Perelman School of Medicine at the University of Pennsylvania, Philadelphia; 3 Leonard Davis Institute of Health Economics, University of Pennsylvania, Philadelphia

## Abstract

This cross-sectional study uses publicly available, nonpartisan data to evaluate trends in lobbying expenditures across health care industries.

## Introduction

Lobbying activities in the US health care sector have drawn increasing public scrutiny over concerns that some firms may be wielding an outsized influence on policy making. However, little is known in the health policy literature about the amount spent on health care lobbying outside the pharmaceutical and health products industry.^1^ Other health care stakeholders, such as hospitals and insurers, have faced regulatory scrutiny and may be investing in lobbying activities to represent their interests.^2^ We evaluated trends in lobbying expenditures for 2000 through 2020 across various health care industries.

## Methods

In this cross-sectional study, we obtained information on health care sector federal lobbying activities for 2000 through 2020 from OpenSecrets, a nonprofit, nonpartisan organization that tracks money in politics.^3^ OpenSecrets collects all lobbying reports filed with the Senate Office for Public Records. Lobbying firms must register and report activities for each client for whom quarterly spending exceeds $3000; organizations employing in-house lobbyists must register and report whether their quarterly spending exceeds $12 500.^4^ The eMethods in the Supplement provides additional details about OpenSecrets data. The institutional review boards of Weill Cornell Medical College and Perelman School of Medicine at the University of Pennsylvania deemed this study to be non–human participant research and waived the requirement for informed consent. The study followed the STROBE reporting guideline.

OpenSecrets identifies the specific industry of each firm within the health care sector. We grouped these classifications into 4 broad categories: pharmaceutical and health product manufacturers, providers (health professionals, hospitals, nursing homes, and associated trade organizations), payers (insurers and health maintenance organizations), and other (health care consultants and policy organizations). The eTable in the Supplement lists additional details on categorization.

We examined trends in lobbying expenditures across the 4 industry categories over time and then evaluated the concentration of spending across deciles of firms within each category. We used the Consumer Price Index to adjust all expenditures to 2020 dollars. Analyses were performed using Stata/SE, version 16.1 software (StataCorp LLC).

## Results

**Figure 1.**
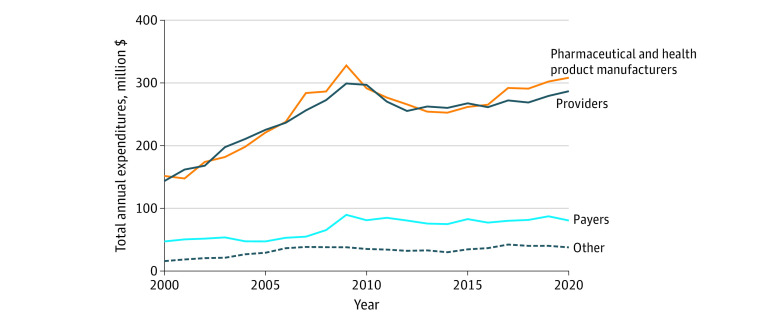
Trends in Federal Lobbying Expenditures Across US Health Care Industries, 2000-2020 All spending adjusted to 2020 dollars using the Consumer Price Index. *Providers* are defined as health professionals, hospitals, nursing homes, and associated trade organizations; *other*, as health care consultants and policy organizations.

**Figure 2.**
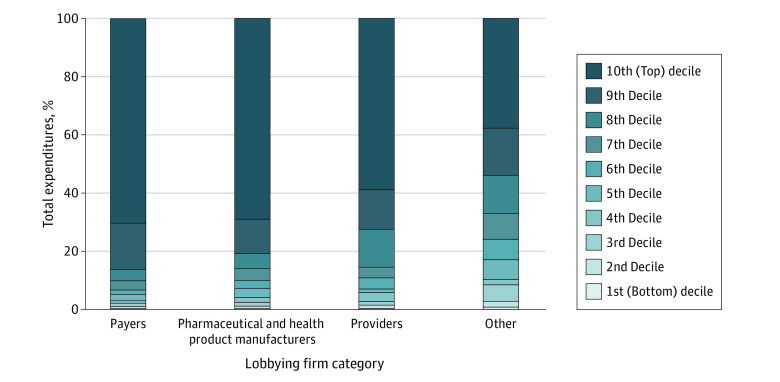
Concentration of Lobbying Expenditures Across US Health Care Industries, 2020 *Providers* are defined as health professionals, hospitals, nursing homes, and associated trade organizations; *other*, as health care consultants and policy organizations.

Lobbying expenditures grew by more than 70% across all 4 categories from 2000 to 2020, with higher levels of growth before 2010 (Figure 1). In 2020, US health care lobbying expenditures totaled $713.6 million vs $358.2 million in 2000. In 2020, pharmaceutical and health product manufacturers spent the most on lobbying activities ($308.4 million), followed by providers ($286.9 million), payers ($80.6 million), and other firms ($37.7 million). Spending was highly concentrated, with the top 10% of firms responsible for 70.4% of spending among payers, 69.0% among manufacturers, and 59.0% among providers (Figure 2). Spending among other firms was less concentrated, with the top 10% responsible for 37.7% of spending.

## Discussion

This cross-sectional study found that lobbying expenditures within the health care sector have increased over the past 2 decades, driven primarily by spending from pharmaceutical and health product manufacturers and providers. Lobbying activities also were not distributed uniformly, with a small number of firms responsible for the majority of expenditures.

This study has limitations. First, given mandated reporting thresholds, the study may not reflect all lobbying expenditures. Second, although the majority of firms only report expenditures associated with federal lobbying, some also include state-level and grassroots lobbying in their mandated disclosures. Third, distinguishing spending by firms with both health care and non–health care lines of business was not always possible. OpenSecrets data, however, have been validated and used for research across multiple sectors of the economy.^5^

These findings reveal that spending from pharmaceutical and health product manufacturers and providers, including their associated trade organizations, comprise the majority of lobbying expenditures from the health care sector. Growth on spending was steeper in the early 2000s relative to more recent years, in part because of lobbying efforts targeting the Affordable Care Act.^6^ A minority of firms spend a disproportionate amount on lobbying, which may lead to certain constituencies being underrepresented in the policy-making process.

## Notes

Supplement. **eMethods.** Details on OpenSecrets Data **eTable.** Health Sector Categorization Based on OpenSecrets Category Codes
eReferences
