# Supplementary material for: Lobbying Expenditures in the US Health Care Sector, 2000-2020
Source: JAMA Health Forum. 2022 Oct 28;3(10):e223801. doi: 10.1001/jamahealthforum.2022.3801 (PMC9617167; doi:10.1001/jamahealthforum.2022.3801)
Supplement: Supplement. — eMethods. Details on OpenSecrets Data eTable. Health Sector Categorization Based on OpenSecrets Category Codes eReferences [file jamahealthforum-e223801-s001.pdf]

Supplemental Online Content

Schpero WL, Wiener T, Carter S, Chatterjee P. Lobbying expenditures in the US health care sector, 2000-2020. *JAMA Health Forum*. 2022;3(10):e223801. doi:10.1001/jamahealthforum.2022.3801

eMethods. Details on OpenSecrets Data

eTable. Health Sector Categorization Based on OpenSecrets Category Codes

eReferences

This supplemental material has been provided by the authors to give readers additional information about their work.

© 2022 Schpero WL et al. *JAMA Health Forum.*

eMethods. Details on OpenSecrets Data

Overview

OpenSecrets is a non-profit, non-partisan organization that tracks money in politics, including campaign contributions and lobbying activities. It compiles, cleans, and standardizes lobbying disclosure reports that organizations file with the U.S. Senate Office of Public Records. Additional data on lobbying activities come from the Office of the Clerk in the U.S. House of Representatives.

As noted in the manuscript, lobbying firms must register and report activities for each client for whom quarterly spending exceeds $3,000; organizations employing in-house lobbyists must register and report if their quarterly spending exceeds $12,500 (increased to $14,000 beginning January 1, 2021). Organizations must make good-faith efforts to report all spending to the nearest $10,000.

Organizations may make disclosures based on the definition of lobbying indicated in the Internal Revenue Code (IRC) or the Lobbying Disclosure Act (LDA) of 1995. Organizations lobbying on their own behalf have the option of using either definition, while lobbying firms must use the LDA definition. Although IRC disclosures may include state and grassroots lobbying expenditures, LDA disclosures only include federal expenditures. An analysis of Fortune 500 companies found that most (70%) use the LDA definition and thus limit their disclosures to federal lobbying activities.[1] In addition, using data from six states, the author estimated that about 19% of disclosed spending from Fortune 500 companies that used the IRC definition could be attributed to state lobbying activities.

Calculating Spending

OpenSecrets calculates annual spending as the sum of all quarterly reports in a given calendar year for an organization. When spending falls below the reporting thresholds, OpenSecrets codes the spending as zero. Spending figures for amended disclosure reports generally replace figures from original reports, except when there are obvious inaccuracies in the amended reports, in which cases the original figures are used. When there are discrepancies between the spending reported by an organization and their outside lobbying firm that exceed $10,000, OpenSecrets uses both the client organization and lobbying firm’s reported amounts in calculating totals and reaches out to the relevant filers for confirmation. In other cases where there are clear reporting errors, OpenSecrets reviews the original Senate records and, when necessary, contacts the Senate Office of Public Records and the relevant filers.

The analysis in this manuscript draws directly from the lobbying industry file in OpenSecrets’ Bulk Data Download lobbying dataset, which includes spending by parent-subsidiary organization and year, along with the subsidiary’s industry category, beginning in 1998.[2] The version used in this analysis was last updated on June 9, 2021, and all observations in the dataset were included for 2000 through 2020. Observations with zero spending were dropped and the data were collapsed to the parent level for analysis. In cases where parent organizations had subsidiaries attributed to different industry categories, the parent was assigned the industry category that corresponded with the majority of lobbying spending across health care subsidiaries in a year.

Additional information on OpenSecrets’ methodology can be found on their website, from which these eMethods were adapted.[3]

© 2022 Schpero WL et al. *JAMA Health Forum.*

eTable. Health Sector Categorization Based on OpenSecrets Category Codes

Category Code	Category Code Description	Health Sector Industry
H0000	Health, Education & Human Resources	Firms assigned to Provider, Manufacturer, Payer, or Other categories based on manual review
H1000	Health professionals	Provider
H1100	Physicians	Provider
H1110	Psychiatrists & psychologists	Provider
H1120	Optometrists & ophthalmologists	Provider
H1130	Other physician specialists	Provider
H1400	Dentists	Provider
H1500	Chiropractors	Provider
H1700	Other non-physician health practitioners	Provider
H1710	Nurses	Provider
H1750	Pharmacists	Provider
H2000	Health care institutions	Provider
H2100	Hospitals	Provider
H2200	Nursing homes	Provider
H2300	Drug & alcohol treatment hospitals	Provider
H3000	Health care services	Provider
H3100	Home care services	Provider
H3200	Outpatient health services	Provider
H3300	Optical services (glasses & contact lenses)	Provider
H3400	Medical laboratories	Provider
H3500	AIDS treatment & testing	Provider
H3800	Mental health services	Provider
H5150	Medical schools	Provider
LH100	Health worker unions	Provider
H4000	Health care products	Manufacturer
H4100	Medical devices & supplies	Manufacturer
H4200	Personal health care products	Manufacturer
H4300	Pharmaceutical manufacturing	Manufacturer
H4400	Pharmaceutical wholesale	Manufacturer
H4500	Biotech products & research	Manufacturer
H4600	Nutritional & dietary supplements	Manufacturer
H4700	Pharmaceutical cannabis	Manufacturer
F3200	Accident & health insurance	Payer
H3700	HMOs	Payer
H3900	Health care consultants	Other
JH100	Health & welfare policy	Other

© 2022 Schpero WL et al. *JAMA Health Forum.*

eReferences

1. Clemens AC. All politics is local, but lobbying is federal and local: the validity of LDA data. *Business and Politics*. 2014;16(2):267-289. doi:10.1515/bap-2013-0017

2. Bulk Data Downloads. OpenSecrets. Accessed August 25, 2022. https://www.opensecrets.org/bulk-data/downloads

3. Methodology. OpenSecrets. Accessed August 25, 2022. https://www.opensecrets.org/federal-lobbying/methodology

© 2022 Schpero WL et al. *JAMA Health Forum.*
